# Giant anogenital tumor of Buschke–Löwenstein in a patient living with human immunodeficiency virus/acquired immunodeficiency syndrome: a case report

**DOI:** 10.1186/s13256-022-03339-1

**Published:** 2022-03-18

**Authors:** Mathurin Kowo, Jude-Marcel Nzoume Nsope Mengang, Sylvain Raoul Simeni Njonnou, Emmanuel Armand Kouotou, Paul Jean Adrien Atangana, Firmin Ankouane Andoulo

**Affiliations:** 1 Department of Internal Medicine and Specialties, Faculty of Medicine and Biomedical Sciences, University of Yaounde I, Yaounde, Cameroon; 2 Yaounde University Teaching Hospital, Yaounde, Cameroon; 3 Department of Internal Medicine and Specialties, Faculty of Medicine and Pharmaceutical Sciences, University of Dschang, Dschang, Cameroon; 4 Dschang District Hospital, Dschang, Cameroon; 5 Department of Biological Sciences, Faculty of Medicine and Pharmaceutical Sciences, University of Douala, Douala, Cameroon; 6 Yaounde Central Hospital, Yaounde, Cameroon

**Keywords:** Buschke–Löwenstein tumor, HIV infection, Local excision, Podophyllin

## Abstract

**Background:**

Buschke–Löwenstein tumor is a giant condyloma acuminata infection that is characterized by degeneration, invasion, and recurrence. It is associated with human papilloma virus infection. It develops around the genital and perineal area, sometimes causing a large budding ulcerated lesion. Although human immunodeficiency virus infection is frequent in Africa, there are few descriptions of Buschke–Löwenstein tumor diagnosis and its management. Screening for other sexually transmitted infections must be systematic among these patients.

**Case presentation:**

We report herein the case of a 21-year-old African origin male patient who developed a perineal swelling. Physical examination showed evidence of a huge exophytic tumor made up of budding pinkish vegetations, with serrated crests, a ‘’butterfly wing’’ structure, and a cauliflower-like appearance crowned with centrifugal circinate lesions. Multiple condylomatous lesions of the anal margin were also present. The patient tested positive for human immunodeficiency virus (cluster of differentiation 4 count of 119 cells/mm^3^) and hepatitis B infections. Real-time polymerase chain reaction revealed human papilloma virus-16 and other high-risk human papilloma virus deoxyribonucleic acid. The diagnosis of Buschke–Löwenstein tumor was made on mass biopsy, and the patient underwent multidisciplinary intervention (surgery, podophyllin application, and antiretroviral therapy). Medium-term evolution was, however, fatal due to opportunistic infection.

**Conclusion:**

Buschke–Löwenstein tumor is a rare tumor associated with human immunodeficiency virus infection. It is more frequent in male human immunodeficiency virus-positive patients. There is a need to screen for other sexually transmitted infections. In most cases, the treatment is surgical, in association with local therapies. However, recurrences are common.

## Introduction

Buschke–Löwenstein tumor (BLT) is a giant condyloma acuminata characterized by its degenerative and invasive potential as well as its recurrent nature after treatment [1, 2]. It is associated with human papilloma virus (HPV) infection, which is mostly transmitted sexually. This means of transmission explains why it is frequently associated with sexually transmitted infections, especially HIV, syphilis, justifying systematic screening [3, 4]. HIV infection increases the risk of HPV transmission, causing extensive condylomatous lesions, favoring degeneration to dysplasia and cancer by modifying local and tissue immunity, especially when associated with HPV type 16, and also increases the risk of unfavorable evolution of HPV lesions [5, 6]. We report herein the case of a young adult with HIV–HBV coinfection who developed an HPV-16-associated BLT.

## Case presentation

A 21-year-old single unemployed male patient from African origin presented to our health facility with an asymptomatic margin swelling noticed over the last 6 months. His past medical history was significant for T10 intercostal herpes zoster complicated with postherpetic neuralgia. He frequently practiced unsafe intercourse, and his partner had no anogenital lesions. He denied any anal or homosexual intercourse.

**Fig. 1:**
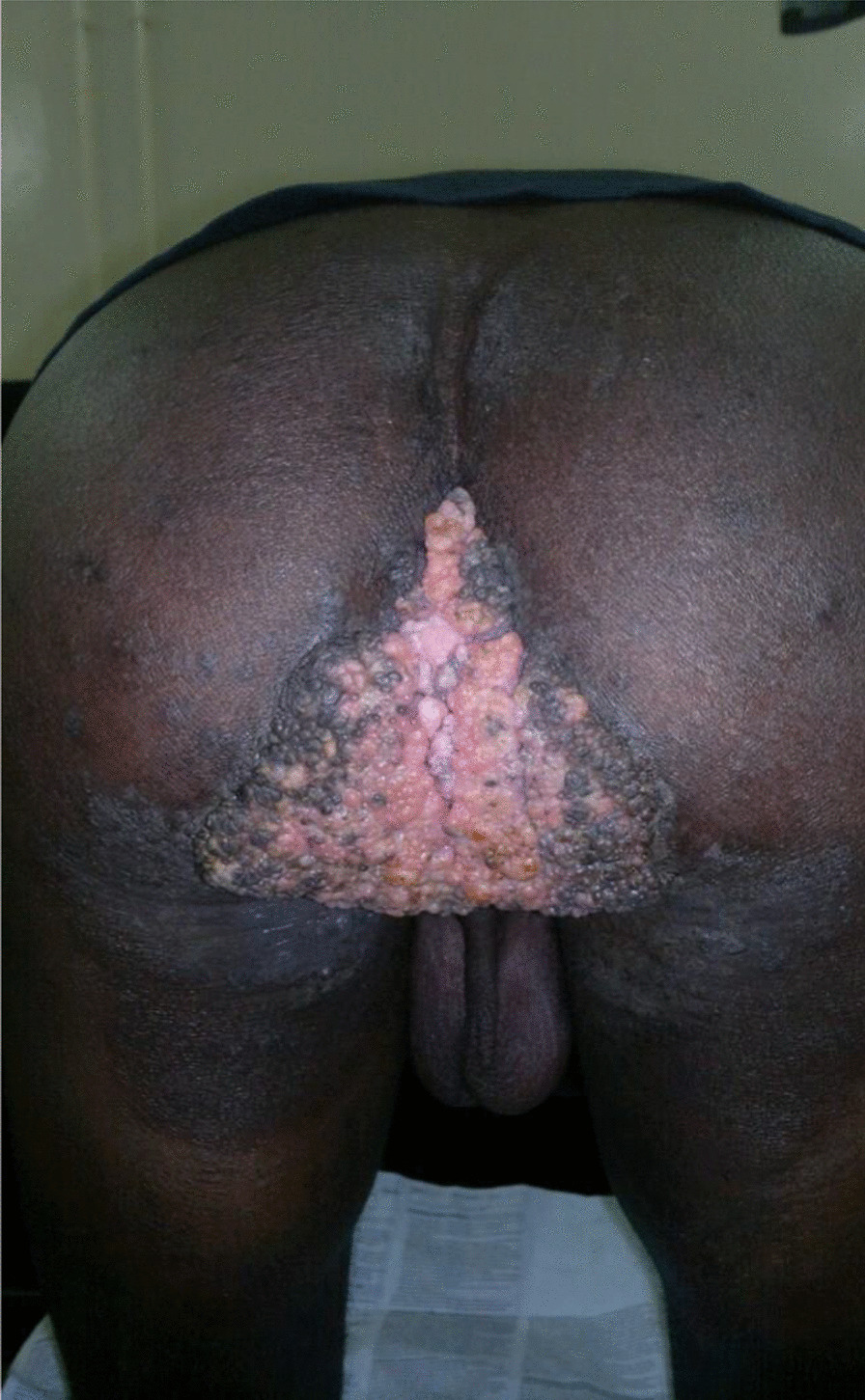
Bulky perianal formation suggestive of Buschke–Löwenstein tumor

Physical examination of the perianal region revealed huge exophytic budding vegetation with batwing disposition, with sharp edges, giving an aspect of a cauliflower crowned with centrifugal circinate lesions, surrounding the anal verge like a sheet (Fig. 1). The glans and body of the penis had multiple scattered condylomatous lesions. The patient was in good general condition and had no palpable lymph nodes.

A complete blood count showed moderate (8.2 g/dL) hypochromic and microcytic anemia (Mean corpuscular volume (MCV) 58 fL, Mean corpuscular hemoglobin concentration (MCHC) 19 pg) with lymphopenia. He tested positive for hepatitis B (HBV viral load of 12 IU/mL) and HIV-1 with CD4 count of 119 cells/mm^3^. Treponema pallidum hemagglutination assay (TPHA)/Venereal disease research laboratory (VDRL), hepatitis D, and chlamydia serologies were negative. Given the lack of financial means, some investigations such as abdominopelvic computed tomography (CT) scan were not performed.

**Fig. 2:**
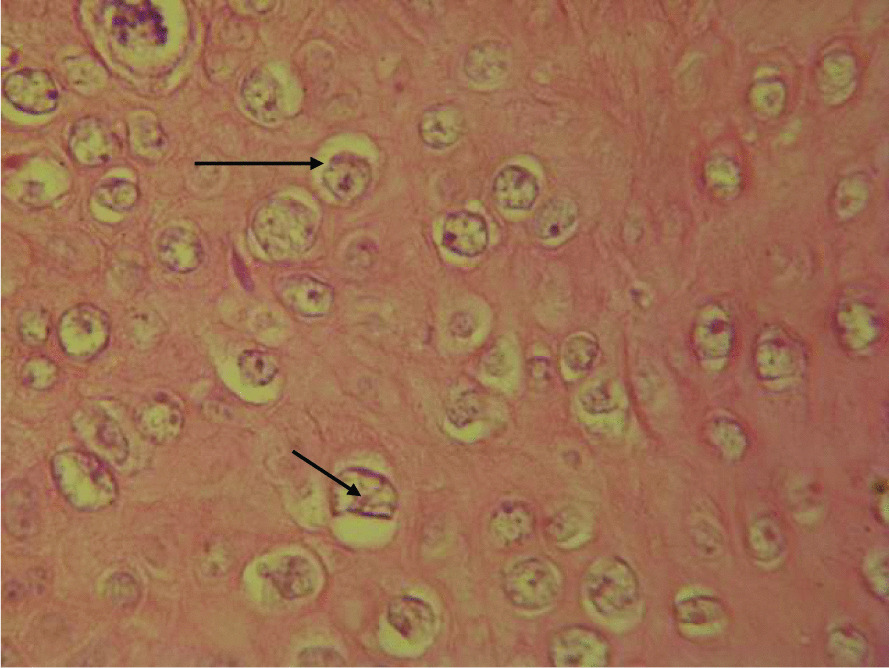
Presence of hyperacanthosis and koïlocytes (in arrow)

**Fig. 3:**
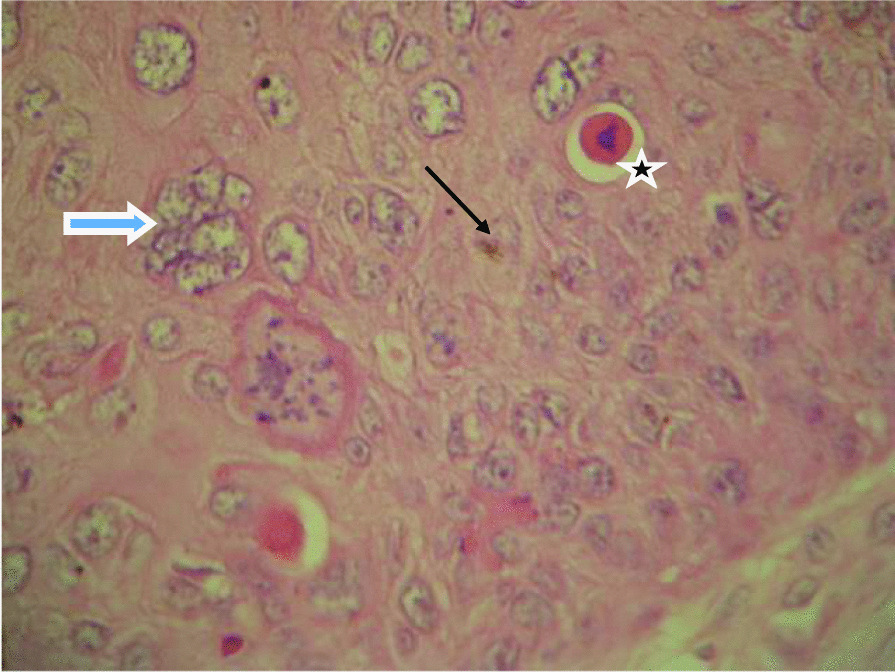
Presence of dysplastic cells (blue arrow), dyskeratotic (star), and mitosis (black arrow)

Histological analysis of a sample obtained from the perianal mass under light microscopy after coloration with hematoxylin and eosin staining showed skin with hyperkeratosis, orthokeratosis and parakeratosis, hyperacanthosis, florid papillomatosis with fusion of papillae. Numerous koïlocytes, characterizing viral infection, were identified inside the acanthosis (Fig. 2). The basal and middle layers had several atypical cells with dyskeratosis. The dermis was inflammatory. These histological findings were consistent with a condylomatous lesion with low-grade dysplasia. HPV-16 DNA was identified by PCR on biopsy proliferation as well as other HR-HPV DNA (Fig. 3). The patient was addressed to surgical consultation 2 months after the first consultation, and highly active antiretroviral treatment (HAART) was started with a single-pill regimen associating tenofovir/lamivudine/efavirenz. Surgical resection of the tumor was performed a month later, and local podophyllin was applied on residual lesions with good outcome. Unfortunately, the patient was not compliant with his treatment and lost to follow-up as soon as he left the hospital. He died 5 months later of neuromeningeal cryptococcosis.

## Discussion

We report the case of a young patient with clinical histological and virologic characteristics of a giant condyloma. The patient was found in the age range with the highest risk of occurrence of HPV infection associated with these tumors. Unprotected sexual intercourse was reported by the patient. The striking finding was the triple coinfection of HBV, HPV, and HIV with severe immunodepression that could justify the extensive nature of lesions with high potential for degeneration and a preexisting low-grade dysplasia. The presence of HR HPV-16 and other HR HPV is consistent with literature that classifies it as the most prevalent in the world and as a high risk. Histopathology of the biopsy sample was consistent with a condylomatous tumor, the presence of significant diffused koïlocytosis all over the epidermis presenting several mitoses and dystrophic cells.

Probably known over the years but well characterized at the start of the twentieth century, BLT is a rare giant condylomatous lesion of the anogenital region. It is more frequent in male immunocompromised patients [7, 8]. Associations with HPV 6 and/or 11 and an absence of secondary localization appear to be arguments for benign tumors [9, 10]. Tumors associated with HPV types 16 and 18 have high degeneration potential due to the oncogenic nature of these viruses [11, 12]. HPV infections are the most frequent sexually transmitted infections and represent an index for sexual activity [13, 14]. This burden leads to vaccination recommendations for teenagers, young females, and males before 26 who have not been vaccinated previously or who have not completed the three-dose series [3].

The peak of occurrence differs by geographic area, being 20–25 years in the USA and Asia and 40–45 years in the Middle East region [4, 12, 13, 15]. HPV type 16 is the most commonly distributed serotype in the world and mostly causes asymptomatic, precancerous lesions and cancers [15, 16]. Identification of HPV can be done on anogenital condylomas by electronic microscopy, immunohistochemistry methods, and biomolecular methods either by hybridization or by PCR. Nevertheless, PCR remains the most sensitive or most commonly used method to detect presence of HPV DNA [3].

A sample biopsy enables us to have histologic confirmation and to look for degeneration foci. BLT is a well-defined tumor. It is characterized under optical microscopy by significant epithelial hyperplasia, sometimes a pseudoepithelioma with an intact basement membrane, hyperacanthosis, hyperpapillomatosis, and koïlocytosis, which are consistent with HPV infection. However, their presence is not constant [17].

Coinfection between HPV and other sexually transmissible infections is frequent and can have a bidirectional impact on the course of the disease, justifying systematic screening. Hence, HPV and HIV coinfection favors progression to dysplasia and cancer by modifying local and tissue immunity [18]. HPV infection stimulates dendritic cells, which are antigen-presenting cells (Langerhans cells) that leave the mucosa by lymph drainage to stimulate lymphocytes, which when activated return to the infected epithelial cells, while HIV infection alters these Langerhans cells. This explains the excessive HPV activity in HIV-infected patients [7, 18]. Coinfected patients have more extensive lesions, which are worst when the CD4 count is less than 200/mm^3^, as in our patient. Persistence, worsening, and recurrence of lesions are frequent in coinfected lesions. It appears that the coinfected population usually has multiple infections by several phenotypes and mostly with high-risk oncogenic viruses HPV 16 and 18 [18]. Despite local aggressiveness, BLT has a lower metastatic potential.

Treatment of anal BLT is primarily surgical. It consists of total excision. However, it is associated with two main problems: recurrence and wound healing delay, often necessitating a temporal loop colostomy [12, 19, 20]. Preventive treatment includes local topicals, electrocoagulation, cryotherapy, and laser destruction of condyloma. These treatments are not very effective in BLT despite some case descriptions. A multidisciplinary approach, as adopted for this patient, is recommended in most cases [12, 21–23]. However, coinfected HPV–HIV patients have a greater risk of failure or recurrence [18]. Fear of stigma and a feeling of shame seemed to be among the most important factors enhancing the evolution of this tumor [24, 25].

## Conclusion

Buschke–Löwenstein tumor or giant condyloma acuminata is a rare tumor associated with HPV infection whose transmission is mainly via the sexual route. It is frequent in males and prompts the search for states of immunosuppression as well as other sexually transmitted infections. The particularity of our patient was a triple coinfection HIV–HPV–HBV and the multidisciplinary management. Although wide surgical excision of the tumor seems to be the optimal therapeutic strategy, local treatment and management of risk factors should also be associated. Early recognition and treatment will provide a good outcome.

## Data Availability

The data used to support the findings of this study are restricted by the direction of the Yaounde University Teaching Hospital in order to protect patient privacy. Data are available from Sylvain Raoul Simeni Njonnou upon reasonable request.
